# Circ_0136666 aggravates osteosarcoma development through mediating miR-1244/CEP55 axis

**DOI:** 10.1186/s13018-022-03303-1

**Published:** 2022-09-15

**Authors:** Xiang Gao, Nanwei Xu, Kaisong Miao, Gao Huang, Yong Huang

**Affiliations:** Department of Orthopedics, Changzhou No.2 People’s Hospital, the Affiliated Hospital of Nanjing Medical University, No.29, Xinglong Lane, Tianning District, Changzhou, Jiangsu China

**Keywords:** Osteosarcoma, Circ_0136666, MiR-1244, CEP55

## Abstract

**Background:**

Accumulating articles demonstrate that circular RNAs play pivotal functions in tumorigenesis. However, the working mechanism of circ_0136666 in osteosarcoma (OS) progression remains to be further clarified.

**Methods:**

Real time-quantitative polymerase chain reaction and western blot assay were applied to determine RNA and protein expression, respectively. Cell proliferation was assessed by 5-Ethynyl-2′-deoxyuridine assay and colony formation assay. Transwell assays were carried out to assess cell migration and invasion abilities. Flow cytometry was performed to analyze cell apoptosis. Cell glycolysis was evaluated by analyzing the uptake of glucose and the production of lactate using the corresponding kits. Dual-luciferase reporter assay and biotinylated RNA-pull down assay were performed to confirm the target interaction between microRNA-1244 (miR-1244) and circ_0136666 or centrosomal protein 55 (CEP55). Xenograft tumor model was utilized to explore the role of circ_0136666 in tumor growth in vivo.

**Results:**

Circ_0136666 expression was prominently elevated in OS tissues and cell lines. Circ_0136666 absence restrained the proliferation, migration, invasion and glycolytic metabolism and promoted the apoptosis of OS cells. Circ_0136666 negatively regulated miR-1244 expression by binding to it in OS cells. MiR-1244 overexpression suppressed the malignant behaviors of OS cells. CEP55 was a target of miR-1244 in OS cells. Circ_0136666 positively regulated CEP55 expression partly by sequestering miR-1244 in OS cells. CEP55 overexpression largely reversed circ_0136666 silencing-mediated influences in OS cells. Circ_0136666 silencing significantly suppressed tumor growth in vivo.

**Conclusion:**

Circ_0136666 silencing inhibited OS progression partly by targeting miR-1244/CEP55 signaling. Silencing circ_0136666 and CEP55 or restoring miR-1244 level might be a potential therapeutic strategy for OS.

## Introduction

Osteosarcoma (OS) is featured by rapid progression and high incidence of lung metastasis [1, 2]. Surgical resection combined with adjuvant or neoadjuvant chemotherapy has notably improved the cure rate of OS patients. Nevertheless, the five-year survival rate of OS patients with metastasis or relapse remains unaffected of about 20% in the last 30 years [3, 4]. Therefore, a detailed understanding of the pathogenesis of OS is needed for OS treatment.

With the rapid development of bioinformatics and sequencing techniques, more and more circular RNAs (circRNAs) are found to be dysregulated in human malignancies [5]. Numerous circRNAs play important regulatory roles in OS development [6–8]. For instance, Zhang et al. found that circ_0136666 contributes to OS development by regulating microRNA-593-3p (miR-593-3p)/ZEB2 axis [9]. Besides, the aberrant upregulation of circ_0136666 and its oncogenic effects in colorectal cancer and breast were also published [10, 11], which attracted our interest. Interestingly, we validated circ_0136666’s high expression in our clinical OS samples. Therefore, we focused on circ_0136666 and investigated its oncogenic roles and mechanisms in OS progression.

CircRNAs regulate cellular biological behaviors by serving as miRNA sponges [12, 13]. It was found that miRNAs were implicated in the regulation of orchestrate proliferation, stromal cell differentiation, osteoarthritis development and OS progression [14–18]. Based on bioinformatics prediction, miR-1244 possessed the potential binding sites with circ_0136666. Yanbin and Jing [19] demonstrated that miR-1244, as the downstream target of circSAMD4A, suppressed the proliferation ability of OS cells. Here, the target interaction between circ_0136666 and miR-1244 and their functional relevance in regulating OS progression were explored.

MiRNAs are involved in the gene regulation by binding to downstream messenger RNAs (mRNAs) [20, 21]. Centrosomal protein 55 (CEP55) was a possible target of miR-1244 based on bioinformatics prediction. CEP55 is a microtubule-bundling protein, and it was initially identified as an important modulator of cytokinesis [22]. Recently, the important role of CEP55 in regulating tumorigenesis has been identified [23–25]. In OS, Xu et al. [26] found that CEP55 promoted OS tumorigenesis by activating AKT signal pathway. In this study, we analyzed the binding relation between miR-1244 and CEP55 and explored their functional association in regulating OS development.

In the current study, we analyzed the expression pattern and biological function of circ_0136666 in OS. The downstream targets of circ_0136666 were predicted by bioinformatics analysis, and rescue experiments were conducted to verify the working mechanism of circ_0136666.

## Materials and methods

### Clinical specimens

A total of 41 pairs of OS specimens and adjacent normal mesenchymal tissue samples were obtained from the metaphyseal regions of long bones of OS patients at Changzhou No.2 People’s Hospital, the Affiliated Hospital of Nanjing Medical University. The adjacent normal tissue samples were ≥ 5 cm away from the edge of OS tissues. The inclusion criteria for OS patients: (1) The clinicopathological diagnosis was confirmed by two pathologists. (2) Patients who had not received radiotherapy, chemotherapy or other treatment before the surgery. The exclusion criteria for OS patients: (1) OS patients who had received radiotherapy, chemotherapy or other treatment before the surgery. (2) Patients who were unsuitable for surgery. (3) Patients who had serious infection. Tissues resected from patients were immediately frozen in liquid nitrogen. Clinical experiment was conducted by the permission of the Ethics Committee of Changzhou No.2 People’s Hospital, the Affiliated Hospital of Nanjing Medical University, and written informed consent was signed by all the patients. The correlation between circ_0136666 expression and clinicopathological characteristics of osteosarcoma patients is shown in Table 1.

**Table 1:** The correlation between circ_0136666 expression and clinicopathological characteristics of osteosarcoma patients

Parameter	Circ_0136666 expression		*P* value^a^
	Low (*n* = 20)	High (*n* = 21)	
*Age (years)*			
< 60	9	12	0.642
> 60	11	9	
*Gender*			
Male	8	8	0.845
Female	12	13	
*Histological grade*			
Low or undifferentiated	15	12	0.381
Middle or high	5	9	
*TNM stage*			
I and II	14	6	0.019*
III and IV	6	15	
*Tumor size*			
≤ 5 cm	15	7	0.018*
> 5 cm	5	14	
*Invasion depth*			
T1 and T2	14	5	0.009*
T3 and T4	6	16	
*Lymphatic metastasis*			
Yes	7	12	0.268
No	13	9	
*Distant metastasis*			
Yes	10	10	0.873
No	10	11	

*TNM* tumor-node-metastasis

^a^Chi-square test; *P < 0.05

### Cell lines

hFOB 1.19, U2OS and SaOS2 obtained from Shanghai Academy of Sciences (Shanghai, China) were cultured with Dulbecco’s modified Eagle’s medium (DMEM) medium (Gibco, Carlsbad, CA, USA) plus 10% fetal bovine serum (FBS, Gibco) and 1% antibiotic mixture (Sangon Biotech, Shanghai, China). hFOB 1.19 cells were maintained at 34℃ with 5% CO_2_, and two OS cell lines were cultivated at 37 ℃ with 5% CO_2_.

### Real-time quantitative polymerase chain reaction (RT-qPCR)

RNA extraction from tissues and cells was performed using Trizol reagent (Invitrogen, Carlsbad, CA, USA). MiR-1244 was reversely transcribed into DNA using stem-loop primer, and miR-1244 level was assessed using the stem-loop primer SYBR Green RT-qPCR Kit (Synbio, Suzhou, China). The reverse transcription of circ_0136666 and CEP55 was carried out using TaqMan Reverse Transcription Reagents (Invitrogen), and the qPCR reaction was conducted using SYBR Green detection reagent (Cowin Biotech, Beijing, China). Primers are listed in Table 2. Glyceraldehyde-3-phosphate dehydrogenase (GAPDH) or U6 was utilized as control for circRNA/mRNA or miRNA. Fold change was analyzed by the 2^−∆∆Ct^ method.

**Table 2:** Specific primers for RT-qPCR

Gene	Primer sequences (5′–3′)
Circ_0136666	Forward primer: GGTGCTCACTGTGCTGAAAA Reverse primer: CAGATGTTCATTGGGTCCAT
MiR-1244	Forward primer: GCCGAGAAGTAGTTGGTTTG Reverse primer: CTCAACTGGTGTCGTGGA
CEP55	Forward primer: GGAGGGCAGACCATTTCAGAG Reverse primer: AGGCTTCGATCCCCACTTAC
U6	Forward primer: CTCGCTTCGGCAGCACA Reverse primer: AACGCTTCACGAATTTGCGT
GAPDH	Forward primer: TATGATGACATCAAGAAGGTGGT Reverse primer: TGTAGCCAAATTCGTTGTCATAC

### RNase R treatment

Total RNA samples were digested with 100 μg/mL RNase R (Applied Biological Materials, Vancouver, Canada) for 20 min at 37℃. The levels of circ_0136666 and its matched linear form protein kinase, DNA-activated, catalytic subunit (PRKDC) were examined by RT-qPCR.

### Cell transfection

The specific small interfering RNA (siRNA) of circ_0136666 (si-circ_0136666), negative control of siRNA (si-NC), the specific short hairpin RNA (shRNA) of circ_0136666 (sh-circ_0136666), sh-NC, mimics of miR-1244 (miR-1244), miRNA NC (miR-NC), inhibitor of miR-1244 (anti-miR-1244), anti-NC, CEP55 re-constructed overexpression plasmid (CEP55) and pcDNA vector (Mock group) were acquired from Genepharma (Shanghai, China) and Sangon Biotech. OS cells were seeded into 6-well plates at the density of 3 × 10^5^ cells/well. Next day, Lipofectamine 3000 (Invitrogen) was utilized to introduce RNA or plasmid into OS cells when cell confluence reached about 70%. After transfection for 6 h, the culture supernatant was replaced by fresh complete medium. After transfection for 24 h, transfection efficiencies were assessed by RT-qPCR and Western blot assay.

### 5-Ethynyl-2′-deoxyuridine (EDU) assay

DNA synthesis was monitored via EDU incorporation using commercial KeyFluor488 Edu Kit (keyGEN Biotech, Jiangsu, China). 4,6-diamino-2-phenyl indole (DAPI) was used to mark cell nucleus. The fluorescence images were captured using the fluorescence microscope (Olympus, Tokyo, Japan). The relative rate of EDU incorporation was analyzed.

### Colony formation assay

OS cells were seeded onto 12-well plates at the density of 200 cells per well. Culture media was replaced every 5 d. After incubation for 2 weeks, cell colonies included more than 50 cells were fixed using 4% paraformaldehyde (Sangon Biotech) and stained by 0.1% crystal violet (Sangon Biotech). The number of colonies was manually counted.

### Transwell assays

In transwell invasion assay, the upper chambers were added with diluted 40 µL Matrigel (1:8; BD Biosciences, San Jose, CA, USA) at 37℃ for 30 min for solidification to pre-coat the upper chambers. In transwell migration assay, un-coated upper chambers were directly utilized for further analysis. A total of 200 μL cell suspension (without serum; in transwell migration assay: 1 × 10^4^ cells; in transwell invasion assay: 8 × 10^4^ cells) was added to the upper chambers, and 10% FBS-added culture medium was pipetted into the lower chambers. FBS acted as chemokine in this experiment. Un-migrated or un-invaded OS cells were wiped out using cotton swab. Migrated or invaded OS cells were dyed using 0.1% crystal violet (Sangon Biotech). Cell number was manually counted using an optical microscope (Olympus, Osaka, Japan) at the magnification of 100×.

### Flow cytometry

After transfection for 72 h, a total of 5 × 10^4^ OS cells were harvested, washed and suspended in binding buffer (BD Biosciences). OS cells were stained by 5 μL Annexin V-fluorescein isothiocyanate (Annexin V-FITC; BD Biosciences) and 5 μL propidium iodide (PI; BD Biosciences) for 15 min. Unstained cell samples and cell samples stained with FITC or PI alone were utilized to determine the threshold. The proportion of apoptotic OS cells (FITC positive, PI positive or negative) was considered as apoptosis rate. Cell samples (1.5 × 10^4^ cells) were loaded onto the FACS CantoII flow cytometer (BD Biosciences), and the apoptosis rate was analyzed by BD FACSDiva software (BD Biosciences).

### Determination of cellular glycolysis

The consumption of glucose and the production of lactate were evaluated using Glucose Uptake Colorimetric Assay kit (Biovision, Milpitas, CA, USA) and Lactate Assay Kit II (Biovision).

### Western blot assay

OS cells were collected and then washed using phosphate-buffered saline buffer (PBS; Sangon Biotech) for three times. Cell lysates were prepared using whole cell lysis buffer (Invitrogen). Protein samples were subjected to 12% sodium dodecyl sulfate–polyacrylamide gel electrophoresis (SDS-PAGE) and dry-transferred onto polyvinylidene difluoride (PVDF) membrane (150 V/2 h; Bio-Rad, Hercules, CA, USA). The non-specific sites in the membrane were sealed using 5% non-fat milk. The diluted primary antibodies of anti-CEP55 (ab170414; Abcam, Cambridge, MA, USA) at the dilution of 1:10,000, anti-hexokinase 2 (HK2; ab227198; Abcam) at the dilution of 1:20,000, anti-pyruvate kinase M 2 (PKM2; ab137852; Abcam) at the dilution of 1:3000 and anti-β-actin (ab8226; Abcam) at the dilution of 1:20,000 were incubated with the membrane overnight. Afterwards, the membrane was labeled with diluted horseradish peroxidase (HRP)-labeled secondary antibody (Abcam) at the dilution of 1:5000. Immunoreactive protein bands were assessed by the enhanced chemiluminescence (ECL) kit (Pierce, Waltham, MA, USA). The quantification of protein bands was performed using Image Lab analysis software (Bio-Rad).

### Subcellular localization

The cytoplasmic and nuclear RNA fractions were isolated using Cytoplasmic and Nuclear RNA Purification Kit (Norgen Biotek, Thorold, Canada).

### Bioinformatics prediction

Circinteractome (https://circinteractome.irp.nia.nih.gov) and TargetScan (http://www.targetscan.org) bioinformatics databases were utilized to predict the interactions between circ_0136666 and miRNAs and between miR-1244 and mRNAs.

### Dual-luciferase reporter assay

To test the interaction between miR-1244 and CEP55 or circ_0136666, dual-luciferase reporter assay was applied to analyze the effect of miR-1244 on the activity of circ_0136666 or CEP55 responsive element. Partial fragment of circ_0136666 or CEP55, including the wild-type or mutant type binding sites with miR-1244, was inserted into pmirGLO vector (Promega, Madison, WI, USA) to generate circ_0136666 WT, circ_0136666 MUT, CEP55 3′ untranslated region (3′UTR) WT and CEP55 3’UTR MUT. OS cells were co-transfected with miR-1244 or miR-NC and luciferase plasmids for 24 h, and the luciferase intensities in different groups were determined by the Dual-Luciferase reporter assay system kit (Promega).

### RNA-pull down assay

MiR-1244 or miR-NC was biotinylated to obtain Bio-miR-1244 or Bio-miR-NC probe, which was subsequently mixed with C-1 magnetic beads (Life Technologies, Carlsbad, CA, USA) to obtain probe-labeled beads. Cell lysates were mixed with probe-labeled beads at 4 ℃ overnight. After the elution, the level of circ_0136666 was determined by RT-qPCR.

### Xenograft tumor model

A total of 10 male BALB/c nude mice (5-week-old) were acquired from Vital River Laboratory Animal Technology (Beijing, China) and then divided into two groups (*n* = 5 in each group). U2OS cells (5 × 10^6^ cells/200 μL PBS) stably transfected with sh-NC or sh-circ_0136666 were subcutaneously inoculated into the right flank of mice. Tumor width and length were measured every week using digital calipers, and tumor volume was calculated as length × width^2^ × 0.5. After injection for five weeks, the mice were killed, and tumors were weighed. Immunohistochemistry (IHC) assay was conducted to analyze the protein level of proliferation marker Ki-67 in tumor tissues using the antibody against Ki-67 (ab15580; Abcam) at the dilution of 1:500. Tumor tissues were utilized to measure the levels of circ_0136666, miR-1244 and CEP55 protein. The protocols in animal experiments were approved by the Animal care Committee of Changzhou No.2 People’s Hospital, the Affiliated Hospital of Nanjing Medical University.

### Statistical analysis

Data were processed using GraphPad Prism 7.0 software (GraphPad, La Jolla, CA, USA). D’Agostino-Pearson omnibus normality test was used to determine the normality of data distribution, and the homogeneity of variances was tested by Levene test. Differences were analyzed by paired or unpaired Student’s *t* test (in two groups) or one-way analysis of variance (ANOVA) followed by Tukey’s post hoc test (in multiple groups). Data were represented as mean ± standard deviation (SD). Linear correlation was analyzed by Pearson’s correlation analysis. The comparisons were considered as statistically significant with *P* < 0.05.

## Results

### Circ_0136666 expression is prominently elevated in OS tissues and cell lines

**Fig. 1:**
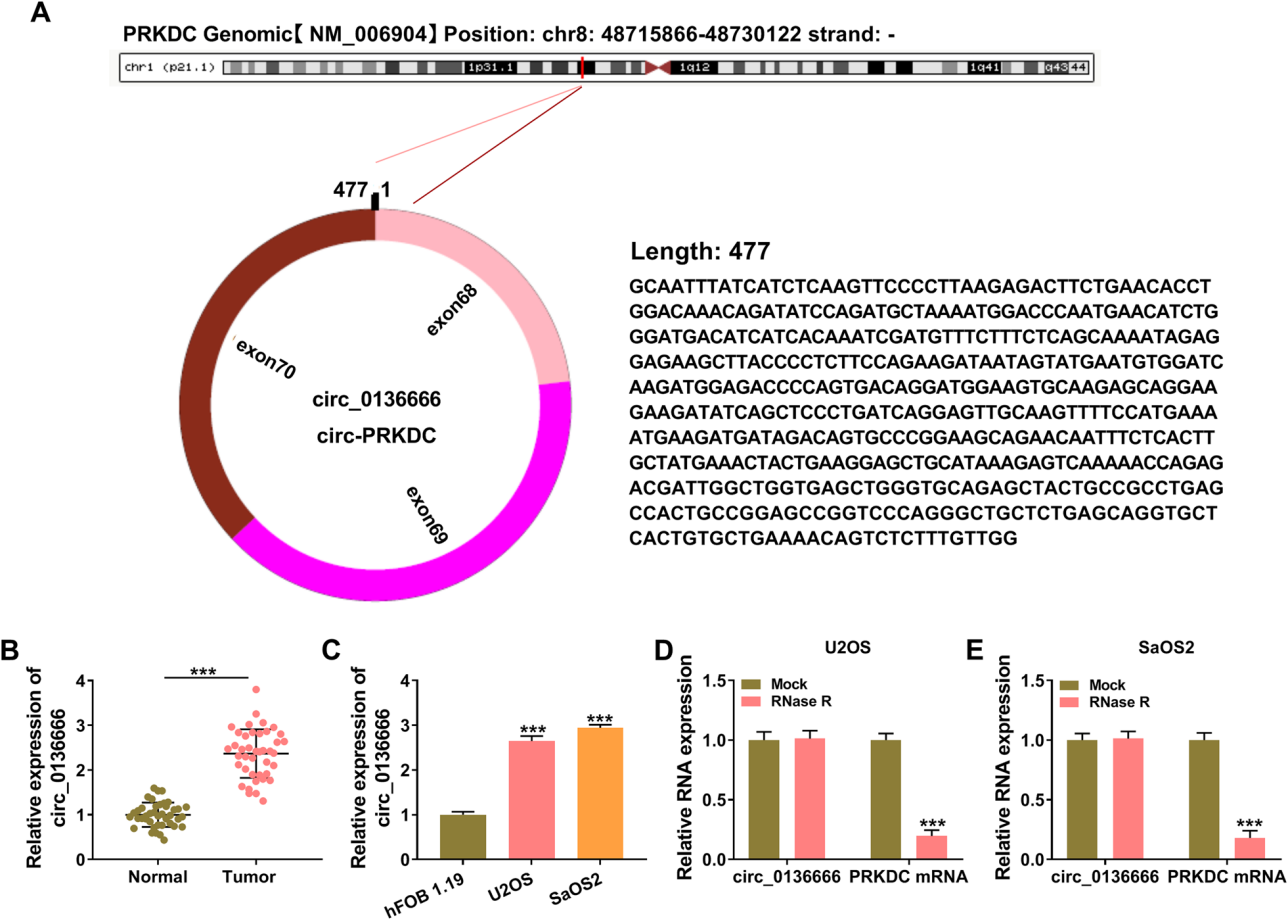
Circ_0136666 expression is prominently elevated in OS tissues and cell lines. **A** Circ_0136666 is derived from the back-splicing of exon 68, 69, and 70 in PRKDC gene. **B** RT-qPCR was applied to determine the expression of circ_0136666 in OS tissues (*n* = 41) and adjacent normal tissues (*n* = 41). **C** The level of circ_0136666 was examined in hFOB 1.19 and two OS cell lines (U2OS and SaOS2) by RT-qPCR. **D** and **E** The RNase R resistance of circ_0136666 and its linear form PRKDC mRNA was analyzed by RT-qPCR. ***P < 0.001

Circ_0136666 (477 nt) is a circular transcript derived from the exon 68, 69, and 70 of PRKDC gene (Fig. 1A). The expression of circ_0136666 was higher in OS tumor tissues (*n* = 41) than that in adjacent normal tissues (*n* = 41) (Fig. 1B). The correlation between circ_0136666 expression and the clinicopathological characteristics of OS patients is summarized in Table 1. High level of circ_0136666 was associated with advanced tumor-node-metastasis (TNM) stage, large tumor size, and increased invasion depth in OS patients (Table 1). We also measured the level of circ_0136666 in human osteoblast cell line hFOB 1.19 and two OS cell lines (U2OS and SaOS2). Compared with hFOB 1.19 cell line, circ_0136666 was highly expressed in both OS cell lines (Fig. 1C). RNase R assay verified that circ_0136666 had better resistance to RNase R degradation than its linear form PRKDC mRNA (Fig. 1D, E). Overall, circ_0136666 might exert an important role in OS progression.

### Circ_0136666 silencing suppresses the malignant properties of OS cells

**Fig. 2:**
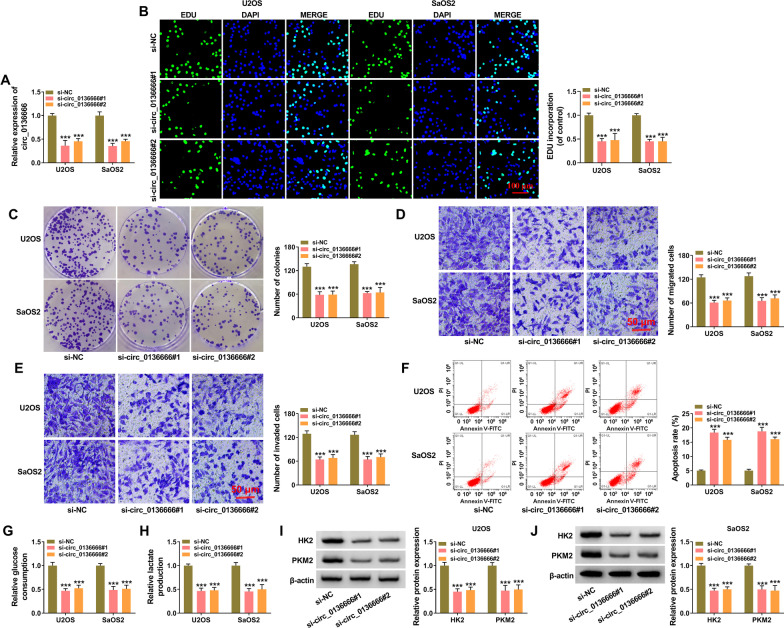
Circ_0136666 silencing suppresses the malignant properties of OS cells. **A**–**J** OS cells were transfected with si-NC, si-circ_0136666#1, or si-circ_0136666#2. **A** RT-qPCR was utilized to analyze circ_0136666 expression in U2OS and SaOS2 cells. **B** Cell proliferation was assessed by EDU assay. **C** Cell proliferation ability was assessed via colony formation assay. **D** and **E** Transwell assays were performed to assess cell migration and invasion abilities. **F** The apoptosis rate (FITC positive and PI positive or negative) was analyzed by flow cytometry. **G** and **H** The consumption of glucose and the production of lactate were analyzed using their corresponding kits. **I** and **J** Western blot assay was performed to measure the protein levels of glycolysis-association markers (HK2 and PKM2) in transfected OS cells. ***P < 0.001

To explore the function of circ_0136666 in OS cells, loss-of-function experiments were performed with si-circ_0136666#1 and si-circ_0136666#2. As shown in Fig. 2A, transfection with si-circ_0136666#1 or si-circ_0136666#2 notably reduced the level of circ_0136666 in OS cells. Circ_0136666 knockdown notably reduced the incorporation of EDU in OS cells (Fig. 2B). The number of colonies was markedly reduced by circ_0136666 silencing relative to si-NC group (Fig. 2C). The results of EDU assay and colony formation assay together suggested that circ_0136666 interference restrained the proliferation of OS cells. Circ_0136666 interference reduced the numbers of migrated OS cells and invaded OS cells (Fig. 2D, E), demonstrating that circ_0136666 interference suppressed cell motility. Flow cytometry was utilized to assess cell apoptosis, and the proportion of OS cells with FITC positive and PI positive or negative was considered as apoptosis rate. Cell apoptosis rate was markedly increased by circ_0136666 knockdown (Fig. 2F), suggesting that circ_0136666 knockdown induced cell apoptosis. Cancer cells change their metabolic phenotype from oxidative phosphorylation to glycolysis, even in the presence of oxygen, which is termed as Warburg effect [27]. The Warburg effect provides growth advantage for cancer cells under hypoxia tumor microenvironment [28]. Subsequently, we explored whether circ_0136666 silencing regulated cell glycolytic metabolism. As shown in Fig. 2G, H, circ_0136666 interference restrained the consumption of glucose and the production of lactate, suggesting that circ_0136666 interference restrained cell glycolytic metabolism. Two glycolysis-associated key enzymes (HK2 and PKM2) were measured in circ_0136666-silenced OS cells. Circ_0136666 knockdown reduced the protein levels of both enzymes (Fig. 2I, J). Taken together, circ_0136666 absence suppressed the proliferation, migration, invasion, and glycolysis and induced the apoptosis of OS cells.

### MiR-1244 is a target of circ_0136666

**Fig. 3:**
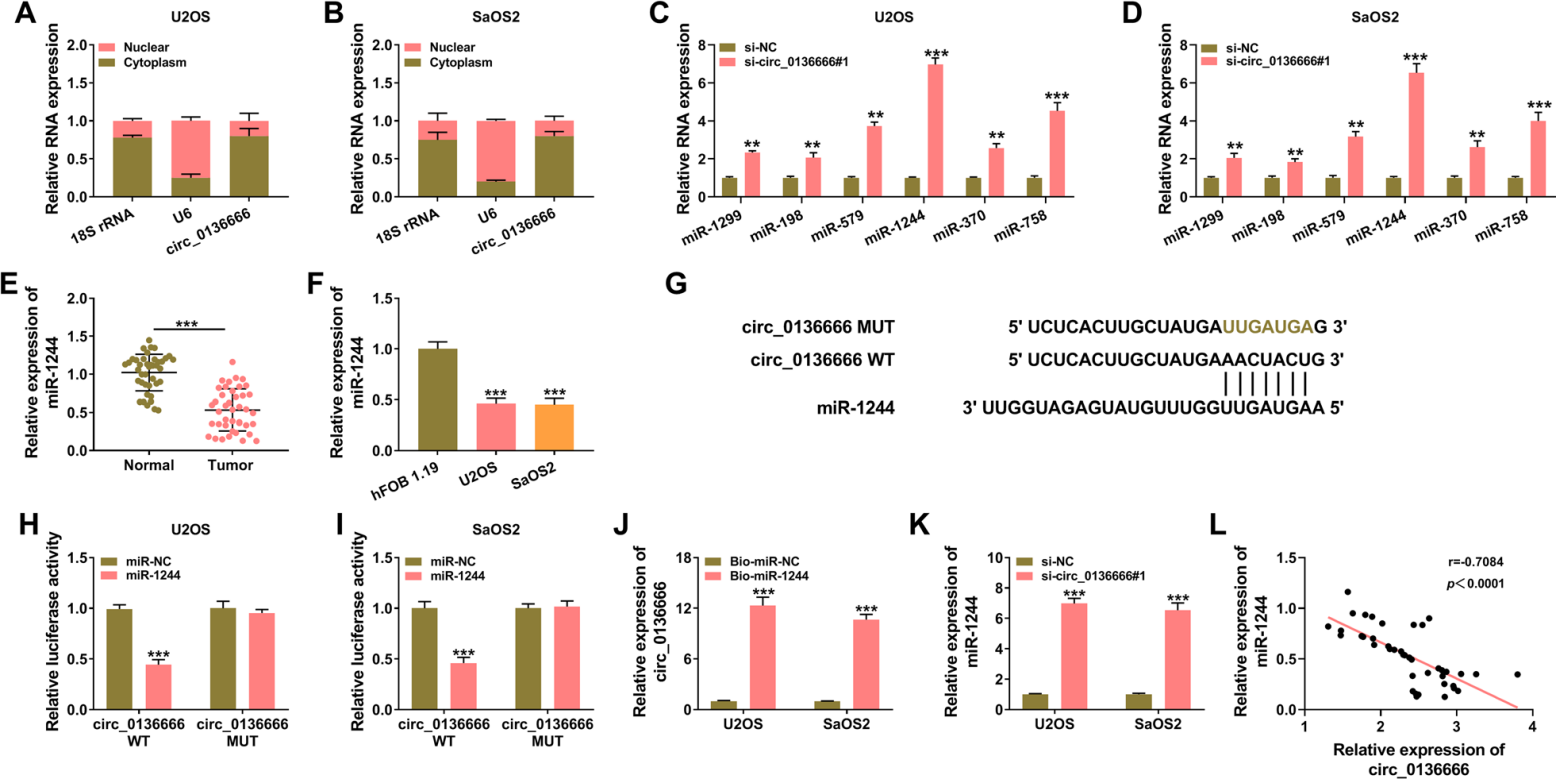
MiR-1244 is a target of circ_0136666. **A** and **B** The subcellular localization of circ_0136666 was analyzed using the commercial kit. **C** and **D** The miRNA targets of circ_0136666 were predicted using bioinformatics software circinteractome, and six candidate miRNAs were screened. RT-qPCR was conducted to measure the expression of six miRNAs (miR-1299, miR-198, miR-579, miR-1244, miR-370, and miR-758) in OS cells transfected with si-NC or si-circ_0136666#1. **E** The level of miR-1244 was determined in 41 pairs of OS tissues and adjacent normal tissues by RT-qPCR. **F** RT-qPCR was conducted to measure the expression of miR-1244 in hFOB 1.19 and two OS cell lines (U2OS and SaOS2). **G** The putative target sequence between circ_0136666 and miR-1244 was shown. **H** and **I** The target relationship between circ_0136666 and miR-1244 was tested by dual-luciferase reporter assay. Circ_0136666 WT or circ_0136666 MUT was co-transfected into OS cells with miR-NC or miR-1244. After transfection for 24 h, relative luciferase activity was determined. **J** Biotinylated RNA-pull down assay was carried out to analyze the target relationship between circ_0136666 and miR-1244 using biotinylated miR-1244 or miR-NC. **K** The expression of miR-1244 in circ_0136666-silenced OS cells was analyzed using RT-qPCR. **L** The linear correlation between the levels of circ_0136666 and miR-1244 was assessed by Pearson’s correlation analysis. **P < 0.01, ***P < 0.001

CircRNAs can act as miRNA sponges to regulate various cellular phenotypes [13]. It was found that circ_0136666 was majorly localized in the cytoplasm of OS cells (Fig. 3A, B), implying its potential to serve as a miRNA sponge. Bioinformatics database circinteractome (https://circinteractome.nia.nih.gov) was utilized to predict the miRNA targets of circ_0136666. Among all the predicted targets, we screened six miRNAs due to their opposite expression pattern or function with circ_0136666 in OS, including miR-1299 [29], miR-198 [30], miR-579 [31], miR-1244 [19], miR-370 [32], and miR-758 [33]. The up-regulation of miR-1244 was the most significant among six miRNAs in circ_0136666-silenced OS cells (Fig. 3C, D). The expression of miR-1244 was notably decreased in OS tissues and cell lines compared with adjacent normal tissues and hFOB 1.19 cell line (Fig. 3E, F). The binding sites between miR-1244 and circ_0136666 predicted by circinteractome are shown in Fig. 3G. Subsequently, dual-luciferase reporter assay and biotinylated RNA-pull down assay were performed to validate the target relationship between miR-1244 and circ_0136666. Transfection with miR-1244 mimics caused a significant reduction in the luciferase activity in circ_0136666 WT group rather than circ_0136666 MUT group when compared with their matching controls (Fig. 3H, I), suggesting that miR-1244 was a target of circ_0136666. MiR-1244 was biotinylated to obtain Bio-miR-1244 to pull down its interacted RNAs. The results revealed that circ_0136666 was enriched when using Bio-miR-1244 (Fig. 3J), suggesting the interaction between circ_0136666 and miR-1244. MiR-1244 expression was notably up-regulated by circ_0136666 silencing in OS cells (Fig. 3K). MiR-1244 expression in OS tissues was negatively correlated with the level of circ_0136666 (Fig. 3L). These results suggested that miR-1244 was a target of circ_0136666 in OS cells.

### MiR-1244 overexpression restrains the malignant behaviors of OS cells

**Fig. 4:**
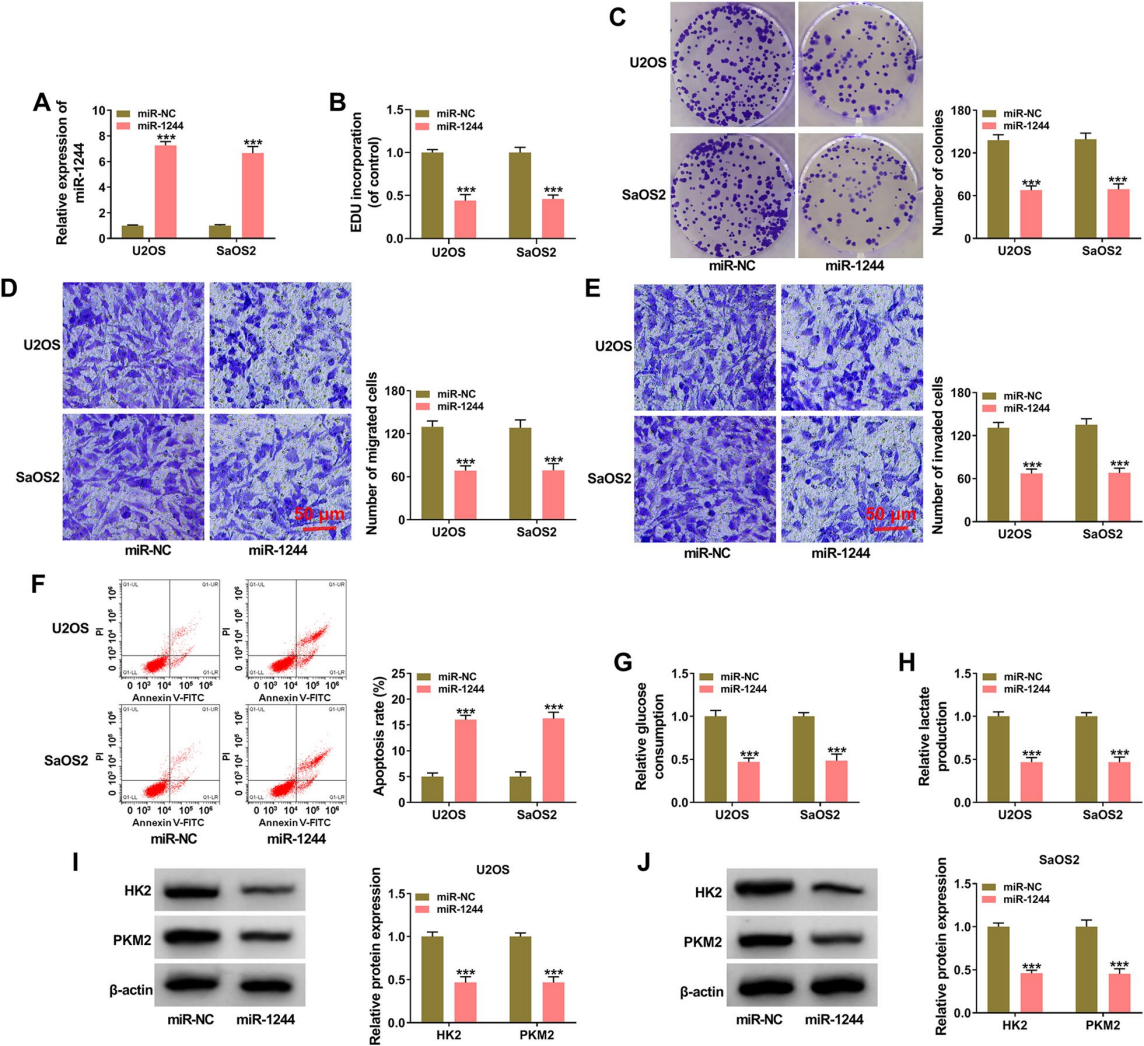
MiR-1244 overexpression restrains the malignant behaviors of OS cells. **A**–**J** U2OS and SaOS2 cells were transfected with miR-NC or miR-1244. **A** miR-1244 expression in transfected cells was assessed via RT-qPCR. **B** EDU assay was applied to determine the proliferation capacity of OS cells. **C** The number of colonies in miR-NC group or miR-1244 group was analyzed via colony formation assay to assess cell proliferation ability. **D** and **E** The numbers of migrated cells and invaded cells were measured via transwell assays to evaluate cell migration and invasion abilities. **F** Cell apoptosis rate was determined via flow cytometry. **G** and **H** Cell glycolytic metabolism was assessed through measuring the uptake of glucose and the production of lactate using their corresponding kits. **I** and **J** The protein levels of HK2 and PKM2 were detected by Western blot assay. ***P < 0.001

Transfection with miR-1244 mimics markedly up-regulated miR-1244 expression in both OS cell lines (Fig. 4A). MiR-1244 overexpression restrained the incorporation of EDU (Fig. 4B), manifesting that miR-1244 overexpression suppressed cell proliferation ability of OS cells. The number of colonies was notably decreased in miR-1244-overexpressed group relative to miR-NC group (Fig. 4C), suggesting that miR-1244 overexpression inhibited cell proliferation ability. MiR-1244 accumulation also repressed the migration and invasion capacities of OS cells (Fig. 4D, E). According to the results of flow cytometry, cell apoptosis rate was significantly increased in miR-1244-overexpressed group relative to miR-NC group (Fig. 4F). MiR-1244 overexpression reduced the consumption of glucose and the production of lactate (Fig. 4G, H), suggesting that miR-1244 suppressed the glycolytic metabolism of OS cells. Also, we found that miR-1244 overexpression decreased the protein expression of HK2 and PKM2 (Fig. 4I, J), which were the key enzymes in cellular glycolysis. These results suggested that miR-1244 exhibited a tumor suppressor role to inhibit the proliferation, migration, invasion and glycolysis and induced the apoptosis of OS cells.

### CEP55 is a target of miR-1244 in OS cells

**Fig. 5:**
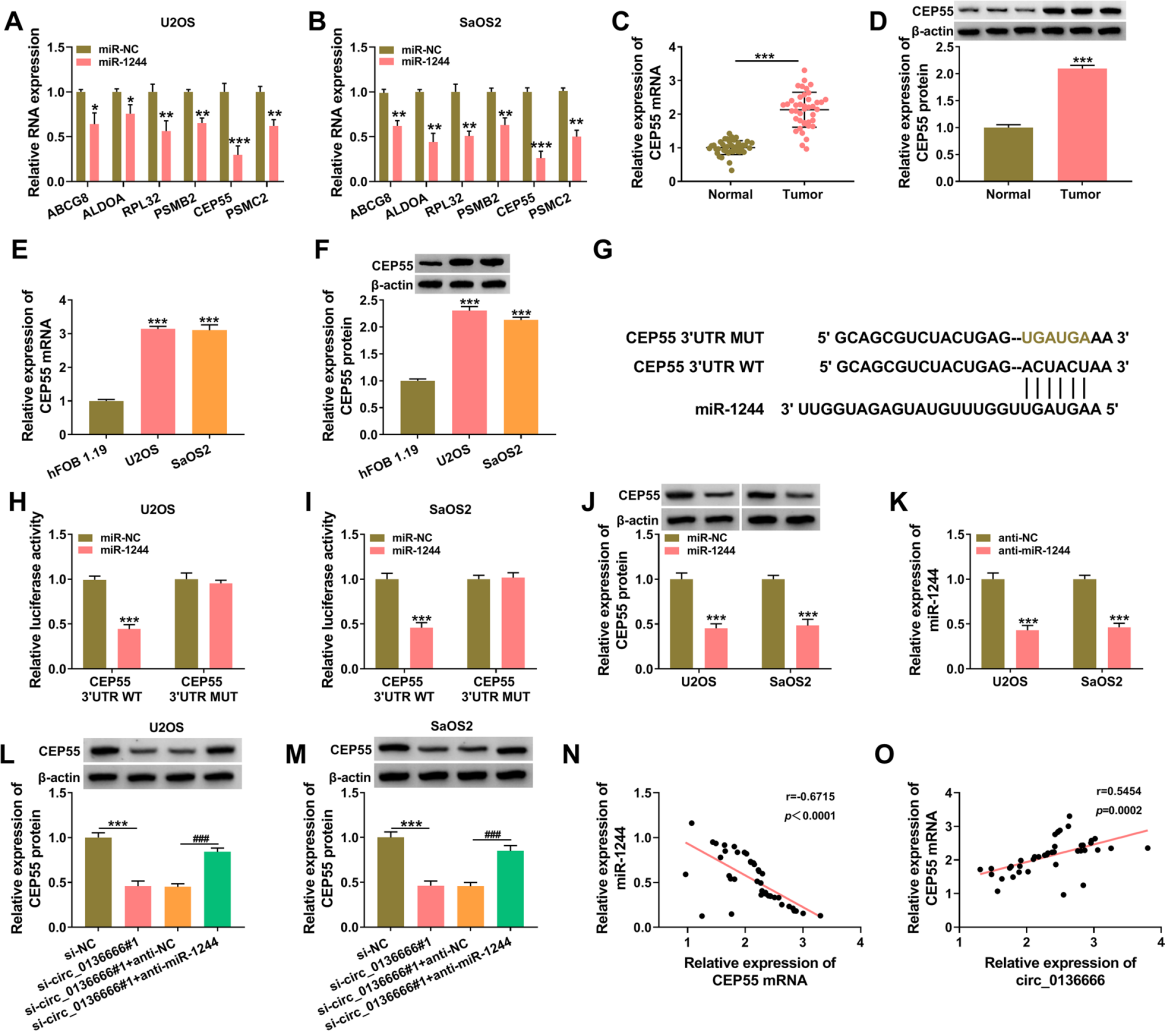
CEP55 is a target of miR-1244 in OS cells. **A** and **B** The mRNA targets of miR-1244 were sought using bioinformatics database TargetScan, and we screened six possible mRNA targets for further study. RT-qPCR assay was performed to analyze the expression of six mRNAs (ABCG8, ALDOA, RPL32, PSMB2, CEP55, and PSMC2) in miR-1244-overexpressed OS cells. **C** and **D** RT-qPCR and Western blot assay were utilized to analyze the expression of CEP55 in OS tissues and adjacent normal tissues in mRNA and protein levels. **E** and **F** The mRNA and protein levels of CEP55 in hFOB 1.19, U2OS and SaOS2 were assessed by RT-qPCR and Western blot assay. **G** The predicted binding sites between miR-1244 and CEP55 were shown. **H** and **I** The interaction between miR-1244 and CEP55 was verified via dual-luciferase reporter assay. **J** The protein level of CEP55 was determined in miR-1244-overexpressed OS cells by Western blot assay. **K** The interference efficiency of anti-miR-1244 in OS cells was analyzed via RT-qPCR. **L** and **M** OS cells were transfected with si-NC, si-circ_0136666, si-circ_0136666 + anti-NC, or si-circ_0136666 + anti-miR-1244. Western blot assay was utilized to measure the protein level of CEP55 in OS cells. **N** and **O** The linear correlation between the expression of CEP55 mRNA and miR-1244 or circ_0136666 was analyzed by Pearson’s correlation analysis. *P < 0.05, **P < 0.01, ***P < 0.001, ^###^*P* < 0.001

miRNAs are implicated in the regulation of gene expression by binding to the 3’UTR of target mRNAs [34]. We wondered whether miR-1244 functioned as a tumor suppressor by targeting mRNAs, and the downstream targets of miR-1244 were predicted by TargetScan database (http://www.targetscan.org). Based on the expression pattern and the tumor suppressor role of miR-1244 in OS, miR-1244-related mRNAs should exhibit high level and oncogenic role in OS. Among the predicted mRNA targets of miR-1244, we screened six mRNAs, including ABCG8 [35], ALDOA [36], RPL32 [37], PSMB2 [38], CEP55 [26], and PSMC2 [39]. After overexpressing miR-1244, the down-regulation of CEP55 mRNA was the most significant in both OS cell lines (Fig. 5A, B). The mRNA and protein expression of CEP55 was markedly up-regulated in OS tissues samples than that in adjacent normal tissues (Fig. 5C, D). Moreover, the mRNA and protein levels of CEP55 were notably elevated in OS cell lines compared with hFOB 1.19 (Fig. 5E, F). The complementary sites between CEP55 3′UTR and miR-1244 are shown in Fig. 5G. Subsequently, dual-luciferase reporter assay was applied to test whether miR-1244 bound to CEP55. U2OS and SaOS2 cells were co-transfected with luciferase plasmid (CEP55 3′UTR WT or CEP55 3′UTR MUT) and miR-1244 or miR-NC. The relative luciferase intensity in CEP55 3′UTR WT group was significantly decreased with the accumulation of miR-1244 (Fig. 5H, I). After mutating the predicted binding sequence in CEP55 3′UTR, the luciferase activity remained unchanged with the co-transfected of miR-1244 or miR-NC (Fig. 5H, I), which verified that miR-1299 bound to the 3’UTR of CEP55 via the predicted sites. MiR-1244 overexpression reduced the protein level of CEP55 in OS cells (Fig. 5J). The results of RT-qPCR verified the high transfection efficiency of anti-miR-1244 in both OS cell lines (Fig. 5K). As shown in Fig. 5L, M, circ_0136666 interference reduced the protein expression of CEP55, while the protein level of CEP55 was largely rescued by the addition of anti-miR-1244. The linear correlation between the expression of CEP55 mRNA and the level of miR-1244 or circ_0136666 was analyzed by Pearson’s correlation analysis. As shown in Fig. 5N, O, it was found that there was a negative correlation between the expression of CEP55 mRNA and miR-1244, and CEP55 mRNA level was positively correlated with the level of circ_0136666. These results demonstrated that CEP55 was a target of miR-1244, and circ_0136666 positively regulated CEP55 expression by sponging miR-1244.

### CEP55 overexpression rescues the malignant behaviors of circ_0136666-silenced OS cells

**Fig. 6:**
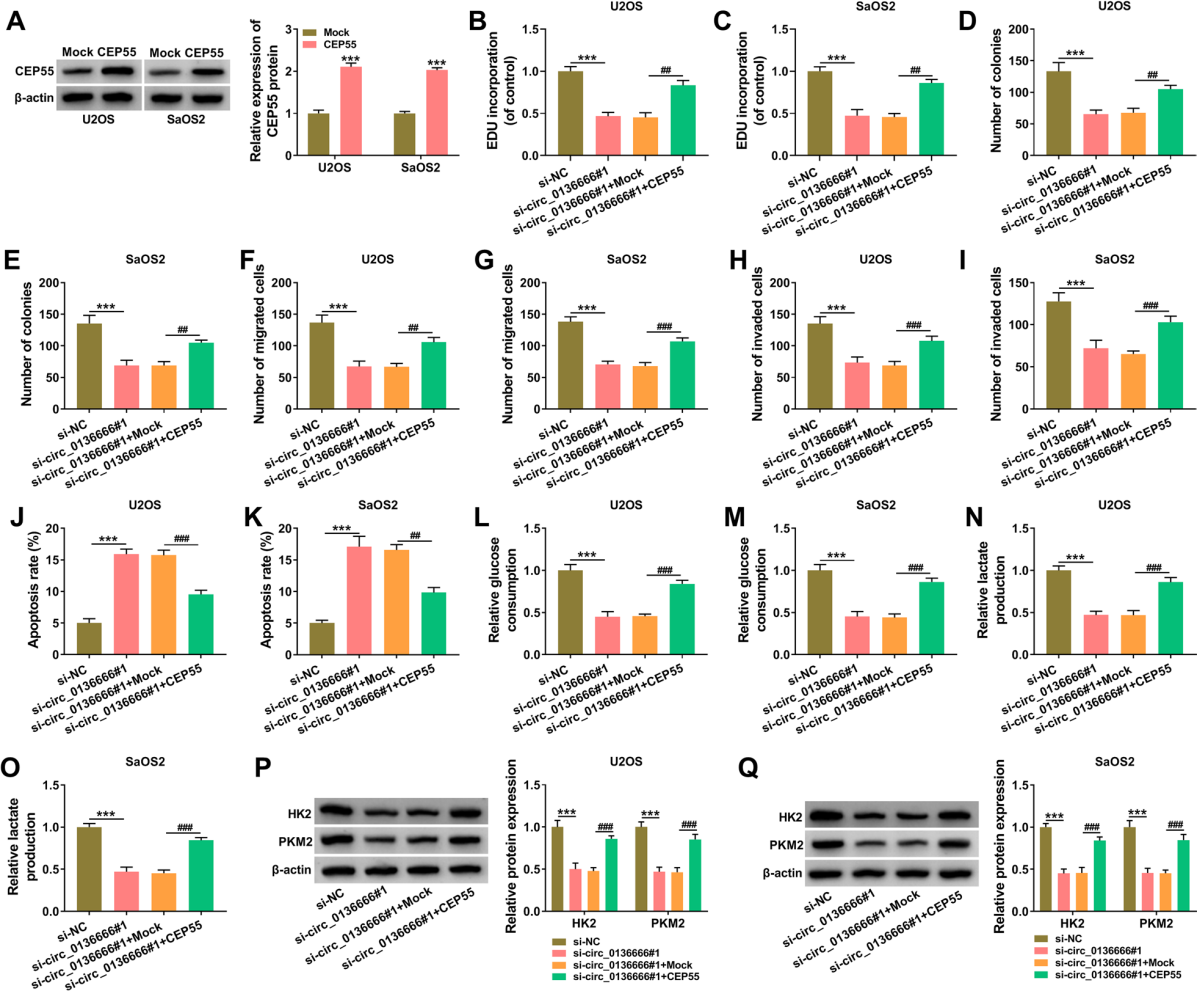
CEP55 overexpression rescues the malignant behaviors of circ_0136666-silenced OS cells. **A** The transfection efficiency of CEP55 plasmid was analyzed by Western blot assay. **B**–**Q** OS cells were transfected with si-circ_0136666 alone or together with CEP55 plasmid. **B** and **C** Cell proliferation ability was determined by EDU assay. **D** and **E** Colony formation assay was carried out to analyze cell proliferation ability. **F** and **G** Cell migration ability was assessed using transwell migration assay. **H** and **I** Transwell invasion assay was conducted to analyze cell invasion ability. **J** and **K** Flow cytometry was utilized to assess cell apoptosis rate. **L**–**O** The consumption of glucose and the level of lactate were analyzed using their matching kits. **P** and **Q** Glycolysis-associated proteins (HK2 and PKM2) were measured by Western blot assay. ***P < 0.001, ^##^*P* < 0.01, ^###^*P* < 0.001

High overexpression efficiency of CEP55 plasmid was verified by Western blot assay (Fig. 6A). To analyze the functional association between circ_0136666 and CEP55, we transfected si-circ_0136666 or together with CEP55 plasmid into OS cells. The addition of CEP55 plasmid largely rescued the proliferation ability of circ_0136666-silenced OS cells as verified by EDU assay and colony formation assay (Fig. 6B–E). Circ_0136666 interference suppressed cell migration and invasion abilities, which were largely reversed by the introduction of CEP55 plasmid (Fig. 6F–I). Circ_0136666 silencing-induced cell apoptosis was largely attenuated by the addition of CEP55 plasmid in OS cells (Fig. 6J, K). CEP55 accumulation also largely rescued the consumption of glucose and the production of lactate in circ_0136666-silenced OS cells (Fig. 6L–O). Circ_0136666 knockdown down-regulated the expression of HK2 and PKM2, and this suppressive effect was largely reversed by the addition of CEP55 plasmid in OS cells (Fig. 6P, Q). Taken together, circ_0136666 silencing blocked the malignant phenotypes of OS cells partly through down-regulating CEP55.

### Circ_0136666 silencing notably suppresses tumor growth in vivo

**Fig. 7:**
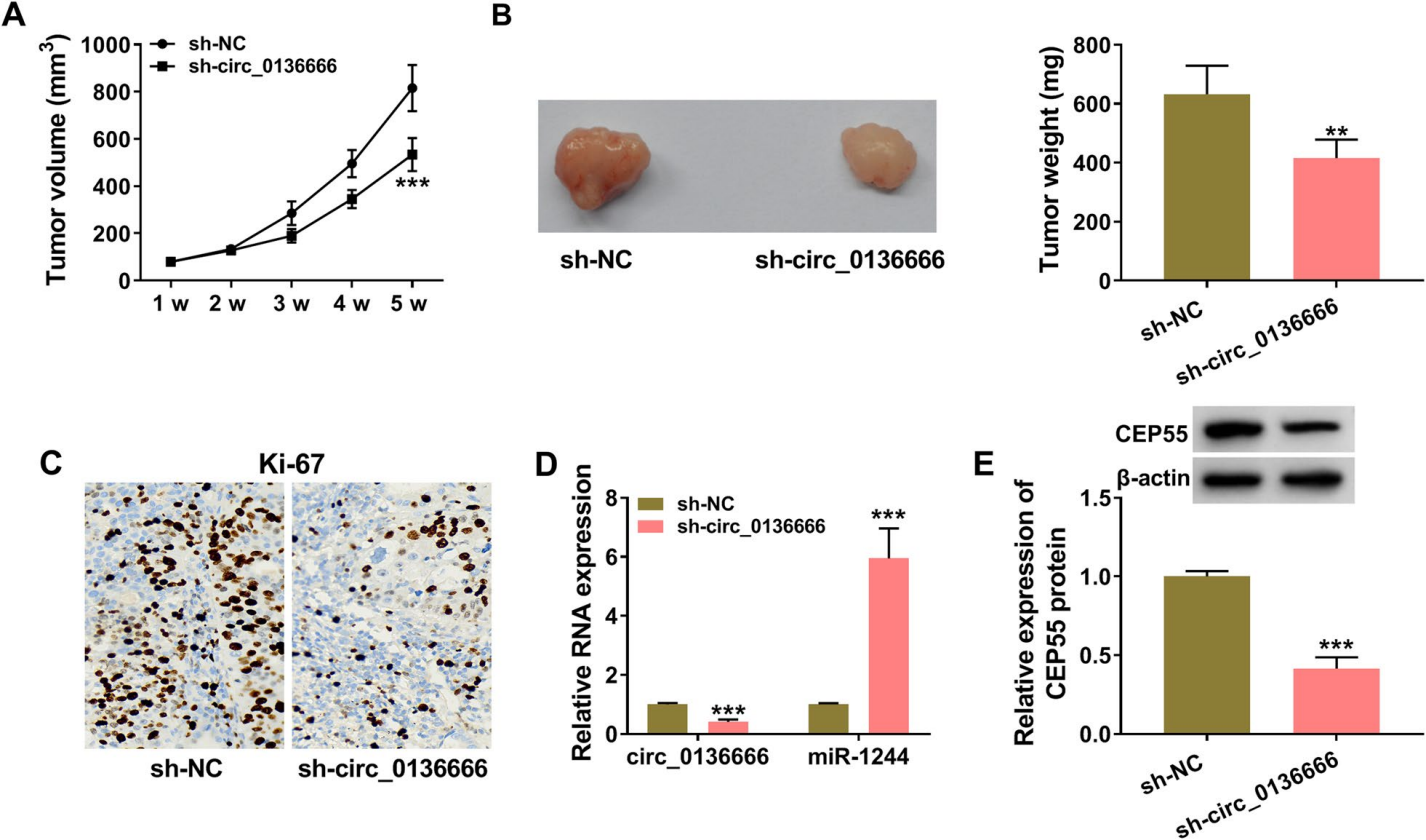
Circ_0136666 silencing notably suppresses tumor growth in vivo. **A** Xenograft tumors were formed through subcutaneously injecting 5 × 10^6^ U2OS cells into the mice, and tumor volume was evaluated every week as width^2^ × length × 0.5. **B** Tumors resected from nude mice after 5-week injection were weighed. **C** The protein level of Ki-67 was examined by IHC assay. **D** and **E** RT-qPCR and Western blot assay were conducted to measure the expression of circ_0136666, miR-1244 and CEP55 protein. **P < 0.01, ***P < 0.001

Xenograft tumor model was established to analyze the effect of circ_0136666 silencing on tumor growth in vivo. Tumor volume and weight were both decreased in circ_0136666-silenced group (Fig. 7A, B), suggesting that circ_0136666 knockdown restrained tumor growth in vivo. As verified by IHC assay, circ_0136666 interference reduced the level of proliferation marker Ki-67 in tumor tissues (Fig. 7C). The expression of circ_0136666 and CEP55 protein was down-regulated in sh-circ_0136666 group relative to sh-NC group (Fig. 7D, E). On the contrary, the expression of miR-1244 was up-regulated in sh-circ_0136666 group than that in sh-NC group (Fig. 7D). Overall, circ_0136666 exerted an oncogenic role to accelerate tumor growth in vivo.

## Discussion

CircRNAs are a class of abundant, stable, and conserved non-coding RNAs with no or limited protein-coding ability generally [40]. CircRNAs play pivotal regulatory roles in the progression of many malignancies, including OS [41, 42]. For instance, circ_100876 is reported to inhibit the proliferation capacity of OS cells by sponging miR-136 [43]. Circ-TADA2A is reported to accelerate the proliferation and motility of OS cells by up-regulating CREB3 via acting as miR-203a-3p sponge [44]. Circ-FAT1 is reported to facilitate the tumorigenesis of OS by elevating the level of Yes-associated protein 1 via sponging miR-375 [45]. Previous studies have demonstrated the oncogenic role of circ_0136666 in certain malignancies, including colorectal cancer [10, 17], breast cancer [11], and OS [9]. Jin et al. [10] found that circ_0136666 contributes to the proliferation ability and invasion ability of colorectal cancer cells through mediating miR-136/SH2B1 signaling. Liu et al. [11] demonstrated that circ_0136666 promotes the development of breast cancer by up-regulating CDK6 via serving as miR-1299 sponge. Zhang et al. found that circ_0136666 is highly expressed in OS, and high level of circ_0136666 initiates OS tumorigenesis by targeting miR-593-3p/ZEB2 signaling [31]. We found that circ_0136666 expression was elevated in OS tissues and cell lines. Circ_0136666 absence suppressed the proliferation, migration, invasion and glycolytic metabolism and promoted the apoptosis of OS cells, demonstrating that circ_0136666 exerted an oncogenic role in OS cells. SiRNAs have been proposed to be used to study the tendon repair process and identify possible therapeutic targets in tendon healing [46]. We thus speculated that circ_0136666-specific siRNAs might be effective therapeutic tools for OS.

Subsequently, the working mechanism behind the pro-tumor role of circ_0136666 in OS development was explored. Circ_0136666 can act as an important regulator in human malignancies by targeting different miRNAs, including miR-593-3p [9], miR-136 [10], miR-1299 [11], and miR-383 [47]. The possible miRNA targets of circ_0136666 were explored by bioinformatics database circinteractome. Subsequently, we demonstrated the interaction between circ_0136666 and miR-1244 by dual-luciferase reporter assay and biotinylated RNA-pull down assay. MiR-1244 expression was reduced in OS tissues and cell lines. In addition, we found that circ_0136666 silencing increased the level of miR-1244 in OS cells. MiR-1244 was identified as a tumor suppressor in several cancers. For instance, Li et al. [48] demonstrated that miR-1244 elevates the sensitivity of cisplatin-resistant NSCLC cells to cisplatin. Liu et al. found that LINC00504 accelerates the malignant behaviors of ovarian cancer cells by down-regulating miR-1244 [38]. In OS, circ-SAMD4A is reported to facilitate OS progression by up-regulating MDM2 via sponging miR-1244 [19], suggesting the tumor suppressor role of miR-1244 in OS. Consistent with the above studies, we found that miR-1244 overexpression inhibited the malignant behaviors of OS cells.

CEP55 was identified as a downstream target of miR-1244 in OS cells. Furthermore, miR-1244 accumulation reduced CEP55 expression in OS cells. CEP55 is an important oncogene in human malignancies. For instance, CEP55 expression is found to be elevated in NSCLC tissues, and high level of CEP55 predicts unfavorable prognosis of NSCLC patients [24]. Yin et al. [25] found that miR-144 inhibits the proliferation and motility of breast cancer cells by reducing CEP55 expression, suggesting the oncogenic role of CEP55 in breast cancer. Yang et al. [49] demonstrated that CEP55 is highly expressed in liver tumor tissues, and high level of CEP55 is associated with dismal prognosis of patients with liver cancer. In OS, Xu et al. [26] found that CEP55 contributes to the proliferation and invasion of OS cells by up-regulating the activity of AKT signaling. In our study, we found that circ_0136666 silencing suppressed the malignant behaviors of OS cells, which were largely reversed by the overexpression of CEP55, suggesting that circ_0136666 knockdown suppressed OS progression partly by reducing CEP55 expression. Circ_0136666 positively regulated CEP55 expression partly by sponging miR-1244 in OS cells. The results of animal experiments suggested that circ_0136666 interference blocked tumor growth, suggesting its oncogenic role in OS in vivo.

**Fig. 8:**
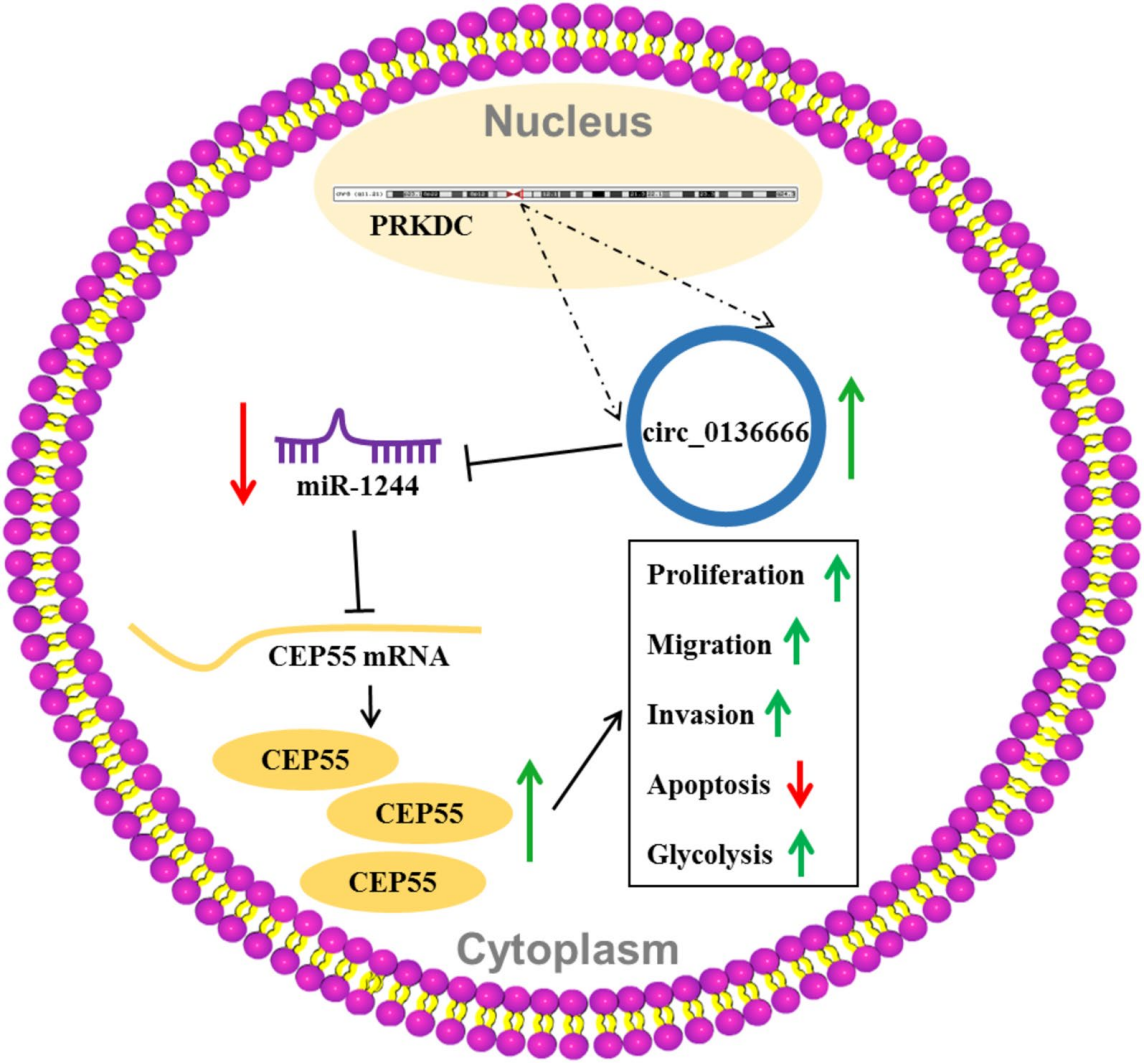
Circ_0136666 contributes to the malignant behaviors of OS cells through mediating miR-1244/CEP55 axis

Taken together, circ_0136666 facilitated the proliferation, migration, invasion and glycolysis and inhibited the apoptosis of OS cells by targeting miR-1244/CEP55 axis (Fig. 8), suggesting that circ_0136666/miR-1244/CEP55 axis might be potential target for OS treatment.

## Data Availability

Not applicable.

## Abbreviations

OS: Osteosarcoma
CEP55: Centrosomal protein 55
GAPDH: Glyceraldehyde-3-phosphate dehydrogenase
PVDF: Polyvinylidene difluoride
ANOVA: Analysis of variance
TNM: Tumor-node-metastasis

## References

[1] He F, Zhang W, Shen Y, Yu P, Bao Q, Wen J, Hu C, Qiu S (2016). Effects of resection margins on local recurrence of osteosarcoma in extremity and pelvis: systematic review and meta-analysis. Int J Surg.

[2] Daw NC, Chou AJ, Jaffe N, Rao BN, Billups CA, Rodriguez-Galindo C, Meyers PA, Huh WW (2015). Recurrent osteosarcoma with a single pulmonary metastasis: a multi-institutional review. Br J Cancer.

[3] Czarnecka AM, Synoradzki K, Firlej W, Bartnik E, Sobczuk P, Fiedorowicz M, Grieb P, Rutkowski P (2020). Molecular biology of osteosarcoma. Cancers (Basel).

[4] Prater S, McKeon B (2020). Osteosarcoma.

[5] Shang Q, Yang Z, Jia R, Ge S (2019). The novel roles of circRNAs in human cancer. Mol Cancer.

[6] Nie WB, Zhao LM, Guo R, Wang MX, Ye FG (2018). Circular RNA circ-NT5C2 acts as a potential novel biomarker for prognosis of osteosarcoma. Eur Rev Med Pharmacol Sci.

[7] Wang L, Wang P, Su X, Zhao B (2020). Circ_0001658 promotes the proliferation and metastasis of osteosarcoma cells via regulating miR-382-5p/YB-1 axis. Cell Biochem Funct.

[8] Zhang H, Yan J, Lang X, Zhuang Y (2018). Expression of circ_001569 is upregulated in osteosarcoma and promotes cell proliferation and cisplatin resistance by activating the Wnt/β-catenin signaling pathway. Oncol Lett.

[9] Zhang C, Zhou H, Yuan K, Xie R, Chen C (2020). Overexpression of hsa_circ_0136666 predicts poor prognosis and initiates osteosarcoma tumorigenesis through miR-593-3p/ZEB2 pathway. Aging (Albany NY).

[10] Jin C, Wang A, Liu L, Wang G, Li G (2019). Hsa_circ_0136666 promotes the proliferation and invasion of colorectal cancer through miR-136/SH2B1 axis. J Cell Physiol.

[11] Liu LH, Tian QQ, Liu J, Zhou Y, Yong H (2019). Upregulation of hsa_circ_0136666 contributes to breast cancer progression by sponging miR-1299 and targeting CDK6. J Cell Biochem.

[12] Hansen TB, Jensen TI, Clausen BH, Bramsen JB, Finsen B, Damgaard CK, Kjems J (2013). Natural RNA circles function as efficient microRNA sponges. Nature.

[13] Panda AC (2018). Circular RNAs act as miRNA sponges. Adv Exp Med Biol.

[14] Chen B, Huang S (2018). Circular RNA: an emerging non-coding RNA as a regulator and biomarker in cancer. Cancer Lett.

[15] Guo J, Liu Q, Li Z, Guo H, Bai C, Wang F (2018). miR-222-3p promotes osteosarcoma cell migration and invasion through targeting TIMP3. Onco Targets Ther.

[16] Liu Q, Geng P, Shi L, Wang Q, Wang P (2019). miR-29 promotes osteosarcoma cell proliferation and migration by targeting PTEN. Oncol Lett.

[17] Giordano L, Porta GD, Peretti GM, Maffulli N (2020). Therapeutic potential of microRNA in tendon injuries. Br Med Bull.

[18] Oliviero A, Della Porta G, Peretti GM, Maffulli N (2019). MicroRNA in osteoarthritis: physiopathology, diagnosis and therapeutic challenge. Br Med Bull.

[19] Yanbin Z, Jing Z (2019). CircSAMD4A accelerates cell proliferation of osteosarcoma by sponging miR-1244 and regulating MDM2 mRNA expression. Biochem Biophys Res Commun.

[20] Esquela-Kerscher A, Slack FJ (2006). Oncomirs - microRNAs with a role in cancer. Nat Rev Cancer.

[21] Muniategui A, Nogales-Cadenas R, Vázquez M, Aranguren XL, Agirre X, Luttun A, Prosper F, Pascual-Montano A, Rubio A (2012). Quantification of miRNA-mRNA interactions. PLoS ONE.

[22] Fabbro M, Zhou BB, Takahashi M, Sarcevic B, Lal P, Graham ME, Gabrielli BG, Robinson PJ, Nigg EA, Ono Y, Khanna KK (2005). Cdk1/Erk2- and Plk1-dependent phosphorylation of a centrosome protein, Cep55, is required for its recruitment to midbody and cytokinesis. Dev Cell.

[23] Tao J, Zhi X, Tian Y, Li Z, Zhu Y, Wang W, Xie K, Tang J, Zhang X, Wang L, Xu Z (2014). CEP55 contributes to human gastric carcinoma by regulating cell proliferation. Tumour Biol.

[24] Jiang C, Zhang Y, Li Y, Lu J, Huang Q, Xu R, Feng Y, Yan S (2018). High CEP55 expression is associated with poor prognosis in non-small-cell lung cancer. Onco Targets Ther.

[25] Yin Y, Cai J, Meng F, Sui C, Jiang Y (2018). MiR-144 suppresses proliferation, invasion, and migration of breast cancer cells through inhibiting CEP55. Cancer Biol Ther.

[26] Xu L, Xia C, Sheng F, Sun Q, Xiong J, Wang S (2018). CEP55 promotes the proliferation and invasion of tumour cells via the AKT signalling pathway in osteosarcoma. Carcinogenesis.

[27] Warburg O (1956). On the origin of cancer cells. Science.

[28] Deberardinis RJ, Sayed N, Ditsworth D, Thompson CB (2008). Brick by brick: metabolism and tumor cell growth. Curr Opin Genet Dev.

[29] Gao AM, Yuan C, Hu AX, Liu XS (2020). circ_ARF3 regulates the pathogenesis of osteosarcoma by sponging miR-1299 to maintain CDK6 expression. Cell Signal.

[30] Lu Z, Wang C, Lv X, Dai W (2021). Hsa_circ_0010220 regulates miR-198/Syntaxin 6 axis to promote osteosarcoma progression. J Bone Oncol.

[31] Wang L, Zhou J, Zhang Y, Hu T, Sun Y (2020). Long non-coding RNA HCG11 aggravates osteosarcoma carcinogenesis via regulating the microRNA-579/MMP13 axis. Int J Gen Med.

[32] Fang C, Wang X, Guo D, Fang R, Zhu T (2020). Circular RNA CircITGA7 promotes tumorigenesis of osteosarcoma via miR-370/PIM1 Axis. Comput Math Methods Med.

[33] Liu W, Long Q, Zhang L, Zeng D, Hu B, Zhang W, Liu S, Deng S, Chen L (2021). Long non-coding RNA X-inactive specific transcript promotes osteosarcoma metastasis via modulating microRNA-758/Rab16. Ann Transl Med.

[34] Ha M, Kim VN (2014). Regulation of microRNA biogenesis. Nat Rev Mol Cell Biol.

[35] Tan J, Liang H, Yang B, Zhu S, Wu G, Li L, Liu Z, Li L, Qi W, Li S, Lin L (2021). Identification and analysis of three hub prognostic genes related to osteosarcoma metastasis. J Oncol.

[36] Shen Y, Xu J, Pan X, Zhang Y, Weng Y, Zhou D, He S (2020). LncRNA KCNQ1OT1 sponges miR-34c-5p to promote osteosarcoma growth via ALDOA enhanced aerobic glycolysis. Cell Death Dis.

[37] Tsai PC, Breen M (2012). Array-based comparative genomic hybridization-guided identification of reference genes for normalization of real-time quantitative polymerase chain reaction assay data for lymphomas, histiocytic sarcomas, and osteosarcomas of dogs. Am J Vet Res.

[38] Zhou X, Fan Y, Ye W, Jia B, Yang Y, Liu Y (2020). Identification of the novel target genes for osteosarcoma therapy based on comprehensive bioinformatic analysis. DNA Cell Biol.

[39] Li GW, Yan X (2019). Lower miR-630 expression predicts poor prognosis of osteosarcoma and promotes cell proliferation, migration and invasion by targeting PSMC2. Eur Rev Med Pharmacol Sci.

[40] Pamudurti NR, Bartok O, Jens M, Ashwal-Fluss R, Stottmeister C, Ruhe L, Hanan M, Wyler E, Perez-Hernandez D, Ramberger E, Shenzis S, Samson M, Dittmar G, Landthaler M, Chekulaeva M, Rajewsky N, Kadener S (2017). Translation of CircRNAs. Mol Cell.

[41] Liu J, Yang L, Fu Q, Liu S (2020). Emerging roles and potential biological value of circRNA in osteosarcoma. Front Oncol.

[42] Zhang Y, Li J, Wang Y, Jing J, Li J (2019). The roles of circular RNAs in osteosarcoma. Med Sci Monit.

[43] Jin J, Chen A, Qiu W, Chen Y, Li Q, Zhou X, Jin D (2019). Dysregulated circRNA_100876 suppresses proliferation of osteosarcoma cancer cells by targeting microRNA-136. J Cell Biochem.

[44] Wu Y, Xie Z, Chen J, Chen J, Ni W, Ma Y, Huang K, Wang G, Wang J, Ma J, Shen S, Fan S (2019). Circular RNA circTADA2A promotes osteosarcoma progression and metastasis by sponging miR-203a-3p and regulating CREB3 expression. Mol Cancer.

[45] Liu G, Huang K, Jie Z, Wu Y, Chen J, Chen Z, Fang X, Shen S (2018). CircFAT1 sponges miR-375 to promote the expression of Yes-associated protein 1 in osteosarcoma cells. Mol Cancer.

[46] Gargano G, Oliviero A, Oliva F, Maffulli N (2021). Small interfering RNAs in tendon homeostasis. Br Med Bull.

[47] Li Y, Zang H, Zhang X, Huang G (2020). circ_0136666 facilitates the progression of colorectal cancer via miR-383/CREB1 axis. Cancer Manag Res.

[48] Li W, Wang W, Ding M, Zheng X, Ma S, Wang X (2016). MiR-1244 sensitizes the resistance of non-small cell lung cancer A549 cell to cisplatin. Cancer Cell Int.

[49] Yang L, He Y, Zhang Z, Wang W (2020). Upregulation of CEP55 predicts dismal prognosis in patients with liver cancer. Biomed Res Int.

